# A Novel Method of Noninvasive Monitoring of Free Flaps With Near-Infrared Spectroscopy

**Published:** 2017-12-15

**Authors:** Hiroyuki Takasu, Kazunobu Hashikawa, Tadashi Nomura, Shunsuke Sakakibara, Takeo Osaki, Hiroto Terashi

**Affiliations:** ^a^Department of Plastic Surgery, Hyogo Prefectural Nishinomiya Hospital, Nishinomiya, Japan; ^b^Department of Plastic Surgery, Kobe University Graduate School of Medicine, Kobe, Japan; ^c^Department of Plastic Surgery, Hyogo Cancer Center, Akashi, Japan

**Keywords:** flap monitoring, free flap, noninvasive, oxygen saturation, spectroscopy

## Abstract

**Objective:** Thrombosis of a site of anastomosis in microsurgical free tissue transfer can result in tissue necrosis. To salvage potentially failing free flap, various methods of monitoring the viability of tissue have been described. We report a novel method of monitoring free flaps using near-infrared spectroscopy.

**Methods:** After microsurgical operation, we monitored the regional oxygen saturation of the flap with using the In-Vivo Optical Spectroscopy. A total of 57 patients participated in this study.

**Results:** Of 57 cases, arterial insufficiency was detected in 1 case and venous insufficiency was detected in 3 cases. Regional oxygen saturation decreased before the flap color changed to a pale or congestive color. We could salvage these 4 patients by re-exploration.

**Conclusions:** The postoperative monitoring with the In-Vivo Optical Spectroscopy is noninvasive, continuous, reliable, and reproducible. This technique could be one of the best methods for monitoring microsurgical free tissue transfers.

Various methods have been described for the monitoring of transplanted tissue viability after reconstruction. These methods are roughly divided into noninvasive techniques and invasive techniques.^1^^,^^2^ The former include clinical monitoring,^3^ surface temperature monitoring,^4^ surface Doppler monitoring,^5^ high-resolution color-duplex ultrasonography,^6^ laser Doppler flowmeter,^7^ and transcutaneous pO_2_ and pCO_2_ monitoring.^8^ The latter include blood glucose concentration,^9^ intraparenchymatous venous pressure,^10^ tissue pH,^11^ and implantable Doppler probe.^12^ While these methods are effective, they are also cumbersome, have less reproducibility, and are not standardized. Furthermore, either regular or intermittent follow-up visits are needed.

The Oximetry Monitoring System (INVOS 5100 C: Medtronic; Dublin, Ireland) provides real-time monitoring of changes in regional oxygen saturation (rSO_2_) of tissue that is placed directly beneath the sensor. This unique system allows clinicians to measure site-specific oxygen levels rather than requiring them to infer the data from systemic, whole body measures, such as blood pressure and pulse oximetry. Furthermore, this system uses 2 data channels, which allows clinicians to conveniently monitor multiple regions throughout the body. This system has been used widely by cardiovascular surgeons and anesthesiologists especially for monitoring regional cerebral oxygen saturation during cardiac surgery.^13^^,^^14^

Using this system, we measured the regional oxygen saturation of transferred tissue. Herein, we report its application as a noninvasive, continuous, reliable, and reproducible method of monitoring transplanted free flap tissue.

## METHODS

### INVOS 5100C

The In-Vivo Optical Spectroscopy (INVOS) system noninvasively monitors site-specific adequacy of perfusion in the tissue directly beneath its sensors.^15^ This provides real-time data on regional oxygen saturation (rSO_2_). Unlike parameters that measure only venous or arterial blood, INVOS technology includes contributions from a 3:1 ratio, thereby yielding a venous-weighted percent saturation. The INVOS system uses near-infrared light at wave lengths that are absorbed by hemoglobin (730 and 810 nm). Light travels from the sensor's light-emitting diode to either a proximal or a distal detector, thereby enabling separate data processing of shallow and deep optical signals. Data from the surface tissue are subtracted and suppressed, reflecting rSO_2_ in deeper tissues (almost 2 or 3 cm beneath its sensors). This feature makes it possible to measure rSO_2_ of brain tissue under the skull bone. We applied this technology to free flap monitoring.

### Patients and methods

**Figure 1:**
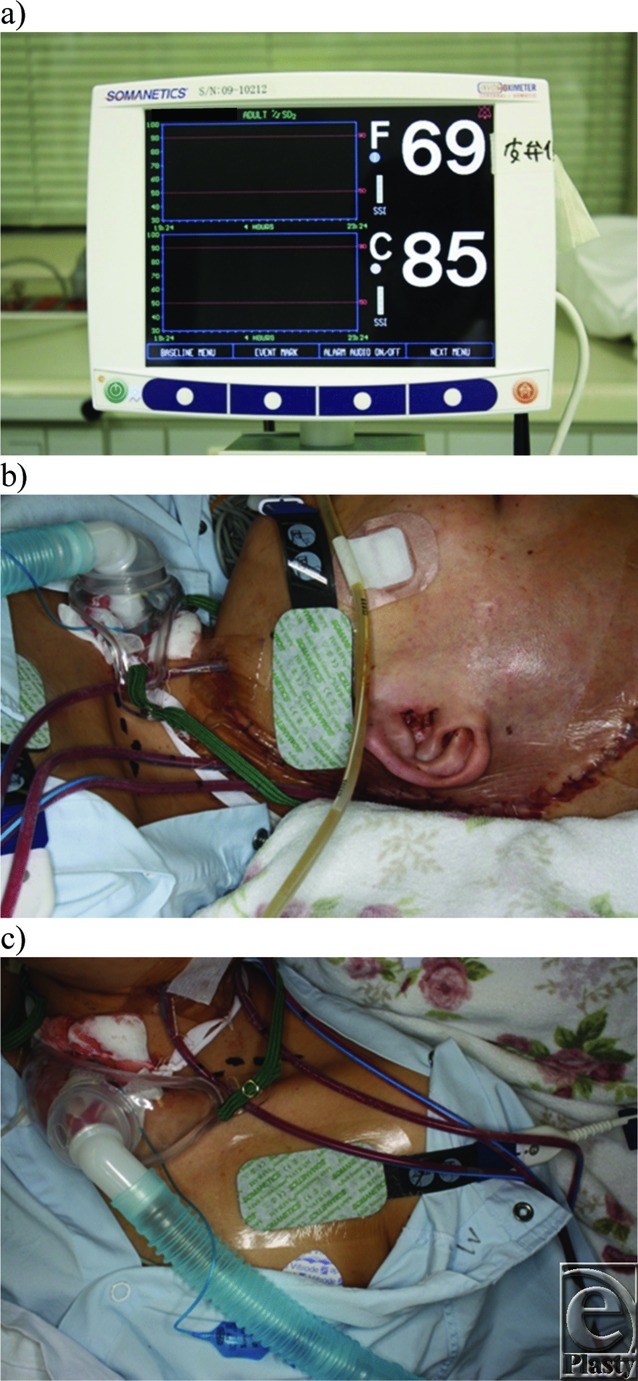
(a) Monitor screen of INVOS system. *F* value indicated flap area rSO_2_ and C value indicated control-area rSO_2_. (b) Sensors to the buried latissimus dorsi flap for skull base reconstruction. (c) Sensors to the anterior part of the chest for control.

Between 2009 and 2017, a total of 57 patients underwent free tissue transfer with monitoring of changes in regional oxygen saturation by INVOS. There were 21 male patients and 36 female patients. The ages of patients ranged from 20 to 82 years, with a mean age of 59 years. After the operation, we threaded the sensors of the INVOS to the flap and a control region. We selected the pectoralis major muscle as the control region and threaded the other sensor to the anterior part of the chest (Figure 1a to c). The specific donor sites used and their distribution among the recipient sites are listed in Tables 1 and 2. This study was approved by Institutional Review Board of Kobe University (approval code: 942).

## RESULTS

In all patients, the monitoring period was 2 to 13 days (median: 6 days). The average flap area rSO_2_ (hereinafter FSO_2_ value) was 73.8 ± 10.6, while the average control area rSO_2_ (hereinafter CSO_2_) was 75.9 ± 10.0 (Table 3). When considered by reconstruction site, the average FSO_2_ was 73.2 ± 9.92 in the head and neck regions, 81.2 ± 7.82 in the breasts, and 66.7 ± 10.1 in the legs (Table 4). When considered by the type of flap used, the average FSO_2_ was 67.8 ± 10.2 for latissimus dorsi flaps, 80.6 ± 7.35 for abdominal flaps, 79.4 ± 7.58 for fibula flaps, and 70.5 ± 6.11 for other flaps (Table 5). During the monitoring period, 1 case of arterial thrombosis and 3 cases of venous thrombosis were observed and reanastomosis was performed. As a result, the flaps survived in all patients.

### Cases 1, 2, and 3

**Figure 2:**
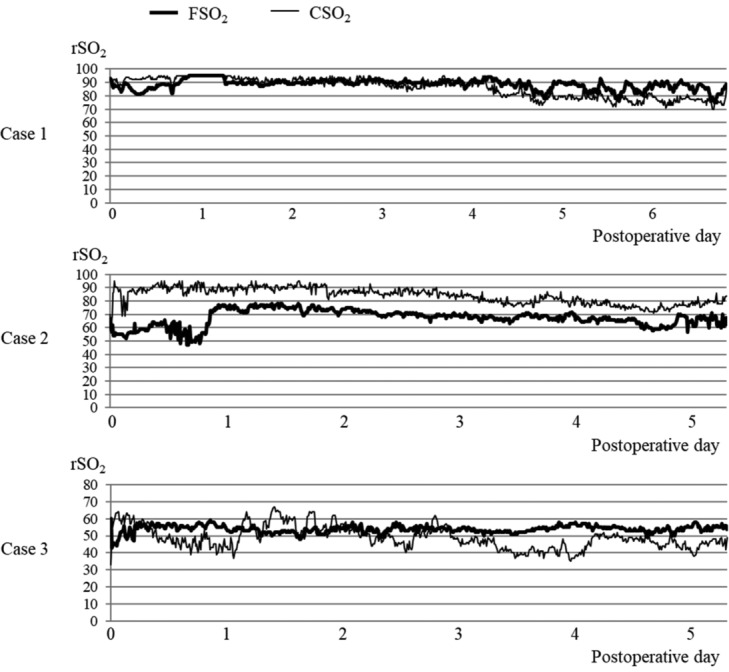
Case 1. A 54-year-old woman after undergoing free deep inferior epigastric perforator flap reconstruction of the mammary region. Case 2. A 66-year-old man after undergoing free latissimus dorsi flap reconstruction of the head. Case 3. A 67-year-old man after undergoing free latissimus dorsi flap reconstruction of the foot. FSO_2_ is plotted by a heavy line, and CSO_2_ is indicated by a thin line. CSO_2_ indicates regional oxygen saturation of control; FSO_2_, regional oxygen saturation of flap; rSO_2_, regional oxygen saturation.

Case 1 was a 54-year-old female patient with breast reconstruction using a deep inferior epigastric perforator flap. Monitoring was conducted for 7 days postoperatively, with an average FSO_2_ value of 88.6 and an average CSO_2_ value of 86.3. Case 2 was a 66-year-old male patient with cranial reconstruction using a latissimus dorsi flap. Monitoring was conducted for 6 days postoperatively, with an average FSO_2_ value of 67.3 and an average CSO_2_ value of 84. Case 3 was a 67-year-old male patient with foot reconstruction using a latissimus dorsi flap. Monitoring was conducted for 6 days postoperatively, with an average FSO_2_ value of 53.8 and an average CSO_2_ value of 48.9. All 3 cases progressed smoothly postoperatively. The postoperative FSO_2_ and CSO_2_ values are shown in Figure 2.

### Case 4

**Figure 3:**
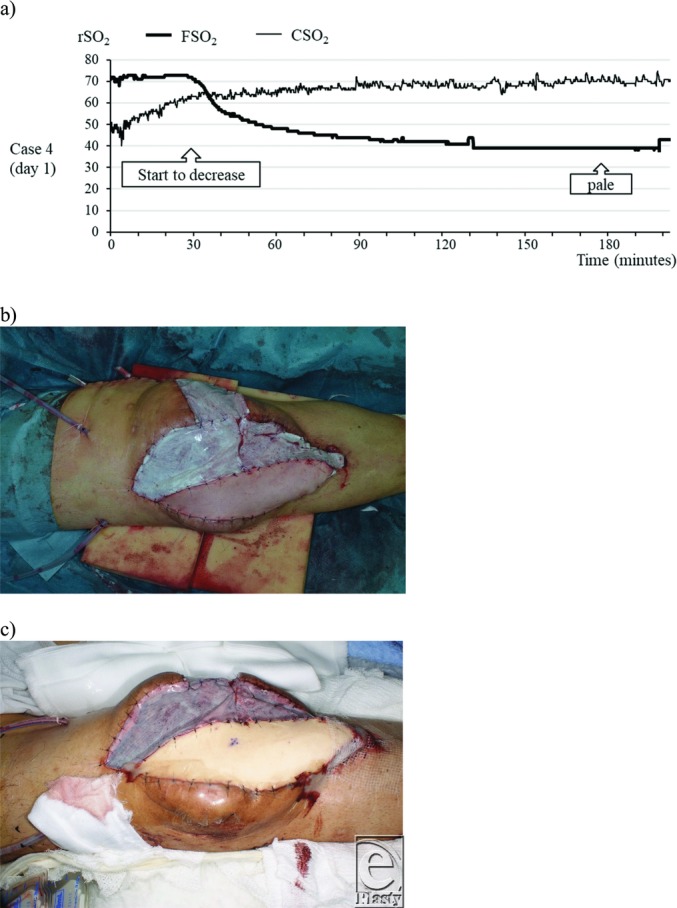
Case 4. A 63-year-old man after undergoing free latissimus dorsi flap reconstruction of the right knee. (a) Within 30 minutes of the patient returning to his hospital room, the FSO_2_ value dropped rapidly over a period of 15 minutes. At that time, no change in flap color was observed. Three hours after the patient was returned to his hospital room, the color of the skin flap was pale and he was diagnosed with arterial occlusion. (b) Photograph immediately after suturing. Artificial dermis was affixed over the muscle flap. (c) Photograph of the pale skin flap. CSO_2_ indicates regional oxygen saturation of control; FSO_2_, regional oxygen saturation of flap; rSO_2_, regional oxygen saturation.

A 63-year-old male patient with arterial occlusion underwent reconstruction surgery with a free latissimus dorsi flap to treat exposure of an artificial joint in the right knee. The descending genicular artery and the great saphenous vein were anastomosed with the thoracodorsal artery and the accompanying vein. Artificial dermis was affixed over the muscle flap and the INVOS was placed over the skin paddle. Thirty minutes after the patient was returned to his hospital room, the FSO_2_ value dropped rapidly from the 70s to the 40s over approximately 15 minutes. At that time, no clear change in flap color was observed. The decline continued slowly after this as well and, when the FSO_2_ value dropped below 40 three hours after the patient was returned to his hospital room, arterial occlusion was diagnosed because of the pale color of the flap, after which arterial reanastomosis was performed. Figure 3a to c shows the postoperative progression of the FSO_2_ value.

### Case 5

**Figure 4:**
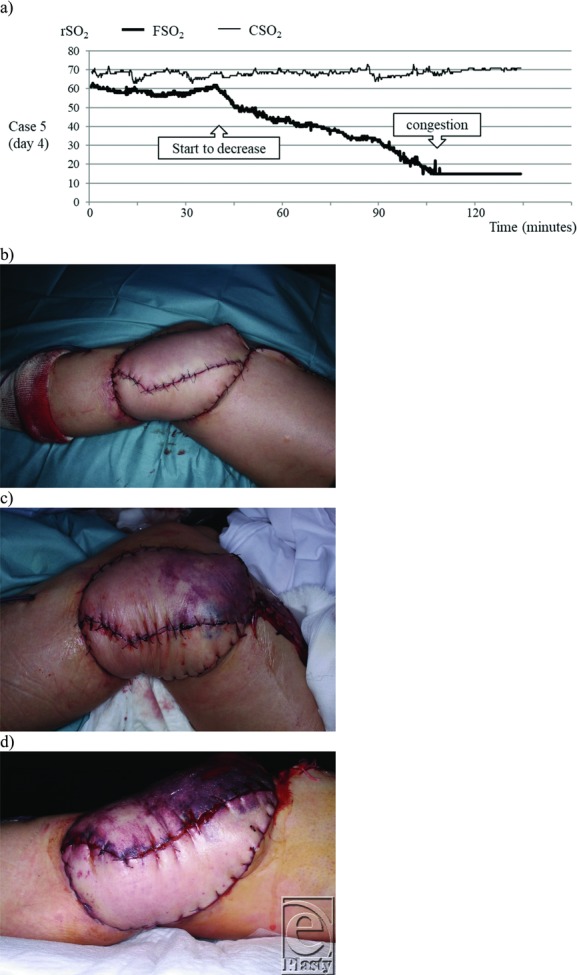
Case 5. A 63-year-old woman after undergoing free latissimus dorsi flap reconstruction of the left knee. (a) The FSO_2_ value dropped over a period of 1 hour on the fourth day postoperatively. A congested color in the skin flap was observed near the time that FSO_2_ reached the minimum value. (b) Immediately after suturing. (c) One-day postoperative findings of congestion were observed on the peripheral side of the divided skin paddle (upper part of this photograph). No abnormal coloring of the skin flap was observed in the center (lower part of this photograph). (d) Four days postoperatively. Since the central skin paddle also presented with a congested color, the cause was deemed to be vein anastomosis-site thrombosis. CSO_2_ indicates regional oxygen saturation of control; FSO_2_, regional oxygen saturation of flap; rSO_2_, regional oxygen saturation.

A 63-year-old female patient with venous occlusion underwent reconstruction surgery with a free latissimus dorsi flap to treat a radiation ulcer in the left knee. The descending branch of the lateral circumflex femoral artery and the accompanying vein were anastomosed with the thoracodorsal artery and the accompanying vein. The skin paddle was divided and sutured with the peripheral side, which was positioned ventrally, and the center, which was positioned dorsally. The INVOS was placed on the central skin paddle. Immediately following the operation, the peripheral skin paddle showed a slight pattern of congestion, which became pronounced 1 day postoperatively. Since the flap color in the central skin island was good, this was deemed to be an intraflap blood flow issue, and the decision was made to monitor the patient's progress. Four days postoperatively, the FSO_2_ began dropping from its previous level of about 60. At that time, no change in flap color was observed. Approximately 1 hour later, the FSO_2_ dropped to 15, the minimum value. Because the central skin island also presented with a congested color at that point, venous occlusion was diagnosed and venous reanastomosis was performed. Figure 4a to d shows the progression of the FSO_2_ value when the venous thrombosis was present.

## DISCUSSION

Baseline variation was observed in both FSO_2_ and CSO_2_, and the amplitude of that variation generally stayed within 20 points. Based on the observation that baseline variation in FSO_2_ and CSO_2_ tends to be linked, it is possible that the INVOS can detect slight changes in the patient's general condition, such as slight fluctuations in interstitial oxygen saturation due to blood pressure and oxygen administration. Although there was a wide range of absolute values for rSO_2_ depending on the patient, when broken down by flap type, rSO_2_ was high in abdominal flaps and low in latissimus dorsi flaps, while a pattern of low rSO_2_ in the legs was observed when broken down by reconstruction site. This led us to consider factors related to blood flow in the flap itself and factors related to blood flow in graft bed blood vessels. However, it is difficult to use the absolute value of rSO_2_ to monitor flap blood flow. Accordingly, it is important to monitor trends that indicate changes in flap blood flow.

Muellner et al^16^ measured rSO_2_ during arterial occlusions with an ischemia model of the brachioradialis muscle that used the INVOS 3100 and tourniquets in healthy adults. This study reported that the rSO_2_ value rapidly dropped from 75 to 25 within 10 minutes of the start of ischemia and, 1 minute after ischemia was corrected, surpassed the preischemia value by rising to 80, after which the value returned to 75. This is believed to be due to the fact that the rSO_2_ value in the INVOS reflects arterial inflow. Specifically, when the rSO_2_ value that dropped because of ischemia rose rapidly after the ischemia was corrected, large amounts of arterial blood continued to flow into the tissue over a short period of time immediately following the release. This resulted in a higher value than the value achieved when the preischemia value was temporarily reached. Ultimately, once the inflow returned to preischemic levels, the rSO_2_ value returned to equilibrium.

In many patients, we observed a pattern in which FSO_2_ increased for a few days following day 1 before achieving equilibrium. This is another indicator of arterial inflow in free flaps following anastomosis. In other words, we inferred that, although flap resistance limits the amount of arterial inflow immediately after anastomosis, an alternative venous return beyond that of the anastomosed veins subsequently formed within a few days of surgery, thereby contributing to a decrease in flap resistance. This ultimately increased arterial blood flow. According to Sakurai et al,^10^ pressure in anastomosed veins tends to gradually decline 3 days postoperatively, while arterial blood flow tends to gradually increase. Our results are consistent with these observations. In particular, we assumed that FSO_2_ indicates the amount of arterial blood flow that enters the flap.

In case 4, where arterial thrombosis occurred, the FSO_2_ value rapidly dropped over approximately 15 minutes, which may demonstrate the fact that arterial inflow decreases rapidly with arterial thrombosis. In addition, although the FSO_2_ value did not bottom out because of thrombus formation, we deduced that the patient's vasculature was not completely occluded and that a small amount of arterial blood continued to flow into the transplanted flap. In contrast, in case 5, where a venous thrombosis occurred, the speed of the decline was slower than the speed of decline in cases of arterial thrombosis, which required approximately 1 hour. This is likely due to the fact that since the expansion of the vascular network inside the flap allows a certain amount of arterial inflow to be accepted even after outflow tracts are blocked by venous thrombosis, the reduction of arterial inflow was milder than in arterial thrombosis. In addition, we inferred that FSO_2_ reached the minimum value because arterial inflow was completely blocked when the flap resistance reached its maximum value, at which time flap congestion was detected clinically. Although findings of flap ischemia are difficult to identify in cases of arterial thrombosis, in both arterial thrombosis and venous thrombosis, clinical changes in flap color seem to first occur when arterial inflow is almost completely blocked. The INVOS system is thought to be a sensitive system for detecting the process of thrombus formation and the progression of decreasing arterial inflow before arterial inflow is completely blocked. This suggests that the INVOS system is a valuable tool that enables the clinical diagnosis of thrombosis at a site of anastomosis and allows for earlier monitoring.

We experienced 2 cases that did not demonstrate vascular thrombosis in spite of a rapid decrease in FSO_2_. In 1 case by suture removal, and in the other case by posture change, FSO_2_ returned to baseline. We considered that the cause of these vascular insufficiencies was tension caused by suture and local pressure, and we believed that we could salvage them before re-exploration by this method.

The characteristics of this system is requirement of threading sensors and measurement of regional oxygen saturation almost 2 or 3 cm beneath its sensors. Consequently, this is especially suitable for buried flap with no skin paddle and can be acceptable in case of skin graft over muscle flap, while it is unfitted for monitoring of shallow region in thin flap or intraoral reconstruction.

Based on these defining characteristics of the INVOS system, we used the FSO_2_ value immediately following INVOS placement as a baseline value in actual clinical settings and directed nurses to contact us if there was a decrease of more than 20 points. Although 1 patient was labeled positive because of a gradual decrease over several days, this drop was likely due to changes in flap hemodynamics (false positive). Otherwise, there were no cases in which flap ischemia or congestion due to anastomosis-site thrombosis went undetected. If a rapid decrease of more than 20 points is positive and the 2 cases that did not demonstrate vascular thrombosis were false positive, the positive predictive value was 66.7% (4/6), the negative predictive value was 100% (51/51), and the number needed to treat is 28.5. In a systemic review by Chen et al,^17^ near-infrared spectroscopy monitoring raised flap salvage rates, lowered flap failure rates, and provided 100% sensitivity and specificity. In our data, specificity was 96% because of 2 false-positive results, although sensitivity was 100%. We infer that if these 2 cases were not monitored by INVOS, then the thrombosis at the site of anastomosis would have occurred later. Therefore, this system has the potential to lower the rate of re-exploration and not only to detect thrombosis quickly.

An implantable Doppler probe is another effective monitoring method, and many reports on its usefulness have been published recently. This device provides a direct and real-time measurement of the flow of vessels and is more efficacious than clinical assessment in terms of flap failure rate, salvage rate, and sensitivity.^18^ Although an implantable Doppler probe is an excellent method for monitoring free flaps, its slight invasiveness comparison with our method could be considered a drawback. There is no perfect device that meets all the ideals for monitoring free flaps. We plan to conduct additional studies that focus on comparing the efficacy of near-infrared spectroscopy with implantable Doppler probes and other flap-monitoring technologies.

## CONCLUSION

Flap blood flow was monitored using a noninvasive mixed blood oxygen saturation monitor. This makes it possible to sensitively detect changes in intraflap blood flow before clinical signs appear, and it is considered useful as a continuous and noninvasive monitor for flap blood flow.

## Figures and Tables

**Table 1:** Types of transferred tissue

Flap type	No. of patients
Latissimus dorsi	26
Deep inferior epigastric perforator and rectus abdominis	22
Fibula	4
Gracilis	1
Groin	1
Radial forearm	1
Anterolateral thigh	1
Scapula	1

**Table 2:** Flap destination

Flap location	No. of patients
Head and neck	33
Breast	13
Lower extremity	11

**Table 3:** Total result

No. of patients	Monitoring period, d	Average FSO_2_	Minimum FSO_2_	Maximum FSO_2_	Average CSO_2_
57	6	73.8 ± 10.6	50.9 ± 16.0	87.9 ± 9.01	75.9 ± 10.0

Abbreviations: CSO_2_, regional oxygen saturation of control; FSO_2_, regional oxygen saturation of flap.

**Table 4:** Flap destination results

Flap location	No. of patients	Average FSO_2_	Minimum FSO_2_	Maximum FSO_2_	Average CSO_2_
Head and neck	33	73.2 ± 9.92	52.8 ± 12.0	88.0 ± 8.38	75.5 ± 7.54
Breast	13	81.1 ± 7.82	60.4 ± 14.2	92.2 ± 5.25	86.2 ± 4.65
Lower extremity	11	66.7 ± 10.1	34.1 ± 16.0	82.5 ± 11.2	65.9 ± 8.65

Abbreviations: CSO_2_, regional oxygen saturation of control; FSO_2_, regional oxygen saturation of flap.

**Table 5:** Flap type results

Flap type	No. of patients	Average FSO_2_	Minimum FSO_2_	Maximum FSO_2_	Average CSO_2_
Latissimus dorsi	26	67.8 ± 10.2	43.7 ± 15.8	83.2 ± 10.5	69.7 ± 9.60
DIEP and rectus abdominis	22	80.6 ± 7.35	59.2 ± 12.6	92.1 ± 4.72	82.2 ± 7.13
Fibula	4	79.4 ± 7.58	49.3 ± 18.4	92.0 ± 3.32	79.1 ± 4.01
Other	5	70.5 ± 6.11	53.4 ± 9.41	90.4 ± 5.82	77.9 ± 6.06

Abbreviations: CSO_2_, regional oxygen saturation of control; DIEP, deep inferior epigastric perforator; FSO_2_, regional oxygen saturation of flap.
